# The efficacy of allograft bone using titanium mesh in the posterior-only surgical treatment of thoracic and thoracolumbar spinal tuberculosis

**DOI:** 10.1186/s12893-020-00793-w

**Published:** 2020-06-12

**Authors:** Bingjin Wang, Wenbin Hua, Wencan Ke, Yukun Zhang, Xianlin Zeng, Cao Yang

**Affiliations:** Department of Orthopaedics, Union Hospital, Tongji Medical College, Huazhong University of Science and Technology, Wuhan, 430022 China

**Keywords:** Allograft, Titanium mesh, Posterior approach, Thoracic and thoracolumbar, Spinal tuberculosis

## Abstract

**Background:**

The bony fusion of allograft bone using titanium mesh in the posterior-only surgical treatment of thoracic and thoracolumbar spinal tuberculosis has not been explained in detail. We aimed to analyze the efficacy of bony fusion of allograft bone using titanium mesh in the posterior-only surgical treatment of thoracic and thoracolumbar spinal tuberculosis.

**Methods:**

We treated 32 thoracic or thoracolumbar tuberculosis patients by one-stage posterior debridement, allograft bone graft using titanium mesh, posterior instrumentation, and fusion from May 2011 to September 2015. The American Spinal Injury Association neurological classification, visual analog scale, and Oswestry disability index scores were analyzed preoperatively, postoperatively, and at final follow-up. The Cobb angles were recorded to evaluate the kyphosis correction and the loss of correction. The bony fusion was evaluated by X-ray and computed tomography images, and the bony fusion classifications were recorded.

**Results:**

All patients had pain relief. The erythrocyte sedimentation rate, C-response protein, and hepatorenal function were normal at final follow-up. The American Spinal Injury Association neurological classification, visual analog scale, and Oswestry disability index scores were improved in all the patients. All patients achieved bone fusion. Twenty-eight patients achieved complete fusion (Grade I), whereas only four patients achieved partial fusion (Grade II). The preoperative Cobb angle was 33.6 ± 9.3°. The Cobb angle was reduced to 10.6 ± 2.6° postoperatively and was found to be 11.4 ± 3.1° at the final follow-up. The mean angle correction was 23.0 ± 8.9°, and the correction rate was 66.2 ± 12.2%. The mean angle lost was 0.8 ± 0.9°, and the lost rate was 5.8 ± 5.4% at the final follow-up.

**Conclusions:**

Allograft bone using titanium mesh in the posterior-only surgical treatment is effective for patients with thoracic and thoracolumbar spinal tuberculosis. It can correct kyphosis, and most patients can achieve complete bony fusion.

## Background

Spinal tuberculosis has a severe global impact on health, especially in developing countries. The complications of spinal tuberculosis include neurologic deficit and kyphotic deformity [1–4].

The primary treatment for spinal tuberculosis is chemotherapy including isoniazid, rifampicin, pyrazinamide, and ethambutol combination therapy for 6–24 months. There is a need to consider surgical treatment for patients having intolerable clinical symptoms, extensive abscess, vertebral collapse, deformity, or spinal cord compression. Moreover, the primary purpose of surgical treatment for spinal tuberculosis is to debride the focus of infection, restore nerve function, and reconstruct spinal stability. The surgical treatments include anterior surgery, posterior surgery, and combined anterior-posterior surgery [5, 6]. However, loss of correction is the major complication of the anterior surgery, due to the quality of the graft and the stability of instrumentation [7–9]. Combined anterior-posterior surgery was developed to address the loss of correction complication. The combined surgery is indispensable for the wide anterior column defects and severe bone destruction that require long segment bone grafting [8]. The posterior approach can reduce the operation time and the intraoperative bleeding, correct kyphosis deformities, and reconstruct the spinal stability for decades [5, 8, 10, 11].

Autograft and allograft have been widely used to reconstruct spinal tuberculosis defects [6, 12–14]. Although the osteoinductive and osteogenetic properties of the allograft are inferior to those of the autograft, the advantages of allograft use is to avoid iliac bone grafting and to preserve osteoconductivity [15, 16]. Moreover, complications of iliac crest bone graft harvesting, such as an increase of surgical time and blood loss, infection, and chronic pain of the donor sites affect patient satisfaction with surgery [17–19]. The application of titanium mesh in the spinal tuberculosis surgery has resulted in the effective reconstruction of bony defects and correction of kyphosis deformities [11, 20, 21]. Moreover, allograft bones with titanium mesh cages achieve favorable clinical results in patients with cervical spinal tuberculosis [22]. However, the bony fusion of allograft bone using titanium mesh in the posterior-only surgery of thoracic and thoracolumbar spinal tuberculosis was not explicitly explained.

The aim of this study is to evaluate the efficacy and feasibility of allograft bone using titanium mesh and the bony fusion in the posterior-only surgical treatment of thoracic and thoracolumbar spinal tuberculosis.

## Methods

### Patient data

We treated 32 consecutive patients with thoracic or thoracolumbar (T12-L1) tuberculosis with one-stage posterior debridement, allograft bone graft using titanium mesh, posterior instrumentation, and fusion from May 2011 to September 2015.

**Fig. 1:**
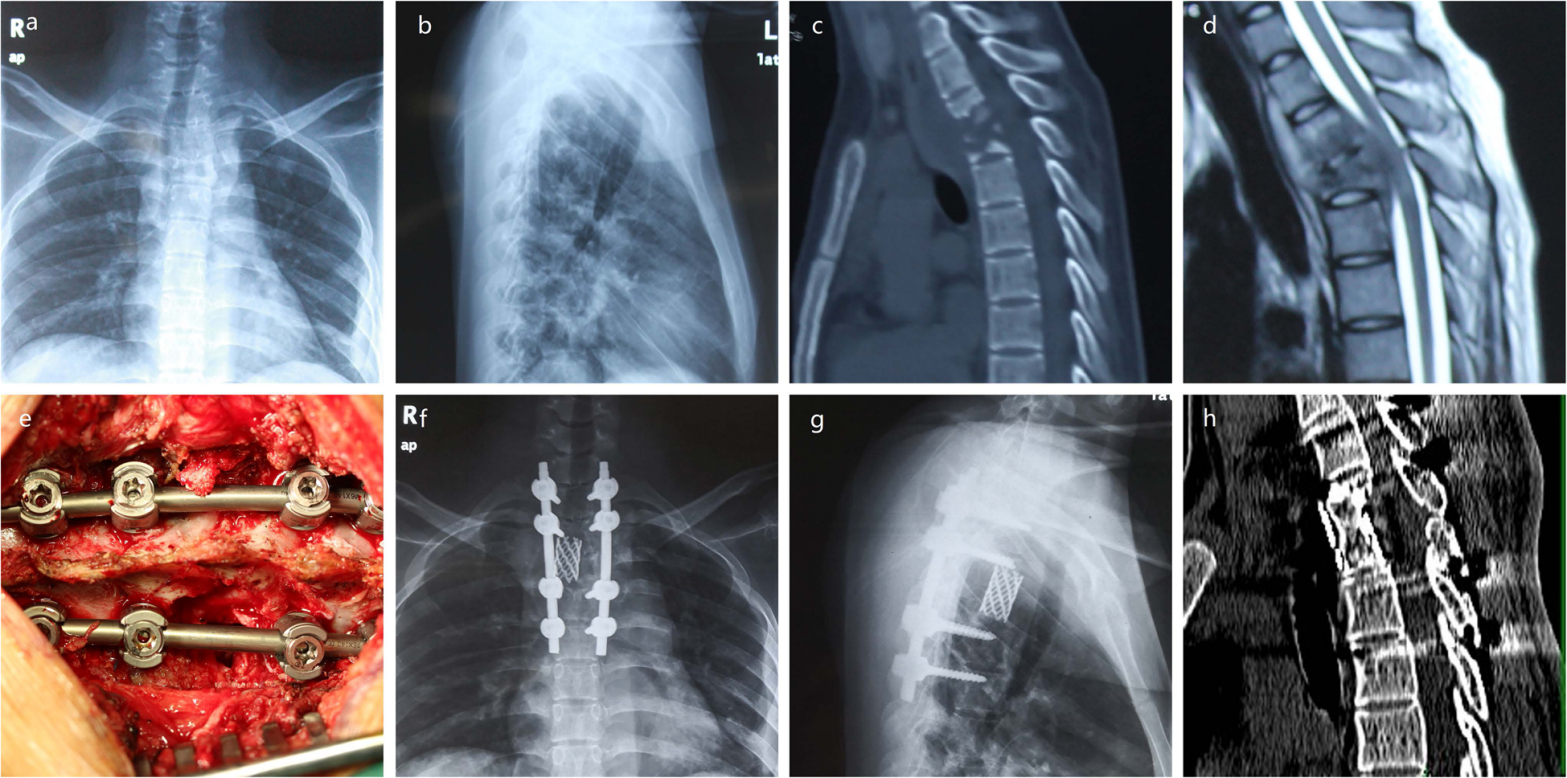
A 26-year-old woman was diagnosed with thoracic tuberculosis after a 6 months history of intermittent back pain and numbness in both lower limbs. **a**-**b** preoperative X-ray showed that T2–3 tuberculosis with the vertebral body destroyed. **c**-**d** preoperative CT and MRI showed T2–3 vertebral body destruction with paravertebral abscess and spinal canal abscess with cord compression. **e** intraoperative image showed one-stage posterior debridement, allograft bone graft using titanium mesh, posterior instrumentation, and fusion. **f**-**g** postoperative X-ray before discharge showed well-positioned titanium mesh and posterior instrumentation. **h** CT scan at final follow-up (5 years) showed good bony fusion, correctly placed titanium mesh, and posterior instrumentation

The spinal tuberculosis diagnosed based on the clinical symptoms, imaging results (anteroposterior and lateral radiography, computerized tomography (CT), and magnetic resonance imaging (MRI)(Fig.1a-d), and hematologic and pathological examinations. The presence of bacillus confirmed the diagnosis. Preoperative or postoperative pathological exams were utilized to make a definite diagnosis. Surgical indications included progressive neurologic deficits, spinal instability, large paravertebral abscess formation, bone sequestration, ineffective conservative treatment or intractable back pain.

The American Spinal Injury Association (ASIA) neurological classification was used to evaluate neurological dysfunction and the visual analog scale (VAS) and Oswestry Disability Index (ODI) scores [23] were used to evaluate pain before surgery, before discharge and at final follow-up. The degree of kyphosis (sagittal Cobb angle) was recorded in the lateral radiograph of full spine preoperatively, postoperatively and at final follow-up to evaluate the kyphosis correction and the loss of the correction. X-ray and CT images were used to assess bony fusion or incorporation of allograft (Fig.1f-h) according to the classification of Tan et al. [24]

### Preoperative management

Anti-tuberculosis drugs (rifampicin, 0.3 g; isoniazid, 0.45 g; ethambutol, 0.75 g) were taken for 2 to 3 weeks before the surgery. Operation was scheduled, when the erythrocyte sedimentation rate (ESR) and C-reactive protein (CRP) were stable or started to decrease and other examination and condition were suitable for operation. The operation was performed immediately in case of aggravated neurological status. The surgical plans were created according to the focus of infection, the involved spinal segment, CT, and MRI. In the patients with evident vertebra damage, two vertebrae above and below the involved segment were fixed.

### Surgical procedure

After inducing general anesthesia, the patients were placed in the prone position with forelimbs held upward. Intraoperative C-arm fluoroscopy was used to position the destroyed vertebras. The mid-spinal incision of appropriate length was made, and the spinous process, bilateral lamina, facets joints, transverse process, or part of the ribs needed to be excised were exposed. Transpedicular screws were placed in the vertebrae according to the preoperative design. After the screws were placed, a temporary rod devoid of any rib excision was placed on the side to avoid movement during the debridement; in addition, it also helped in the placement of titanium mesh. After excision of the part of the transverse process and ribs, the collapsed vertebrae, necrotic disc, and prevertebral or paravertebral abscesses were completely removed. The posterior roots were cut in the patients with the excision of ribs. After the prepared bone trough was cleaned by saline irrigation, a suitable titanium mesh with allogenic bone was inserted into the designed preoperative place to reconstruct the stability. Intraoperative fluoroscopy was used to confirm the position of titanium mesh. The intact posterior fixation instrument was placed. Isoniazid or streptomycin was administered locally, and two drainage tubes were placed before the incision was sutured. The excised vertebrae and necrosis tissues were sent for pathological examination, and the abscess was sent for smear examination.

### Postoperative management

The drain was removed when the drainage flow was < 50 ml per 24 h. All patients were treated with anti-tuberculosis drugs same as given preoperatively for 12 to 18 months. In osteoporosis patients, thoracolumbar brace was continually used for 3 to 6 months postoperatively. All patients were evaluated with physical examination, radiograph (X-ray and CT), ESR, CRP and hepatorenal function at follow-up.

### Statistical analysis

The preoperative and postoperative Cobb angle, VAS and ODI scores were analyzed by ANOVA and *t*-tests (Version 25.0, SPSS, Chicago, Illinois, USA). *P* values < 0.05 were considered statistically significant.

## Results

The mean age of the patients at the time of treatment was 44.1 years (range, 24–72 years) (Table 1). There were 18 male patients and 14 female patients. The mean follow-up was 31.2 months (range, 24–60 months). The regions of spinal tuberculosis included 19 thoracic and 13 thoracolumbar. The mean duration of surgery was 172.7 mins, and the mean blood loss was 710.9 ml (Table 1). Two patients who had suffered the wound infection due to mycobacterium were healed by debridement and antibiotics. All patients experienced pain relief. ESR, CRP, and hepatorenal function were normal at the final follow-up. No patient developed complications related to the spinal instrument, titanium mesh, or allograft.

**Table 1:** Characteristics and clinical data of the patients

Variable	Statistic
Mean age/years	44.1
Gender	
Male	18
Female	14
Region of tuberculosis	
Thoracic	19
Thoracolumbar	13
Mean surgery duration (minutes)	172.7
Mean blood loss (ml)	710.9

Neurological status of all patients was evaluated according to the ASIA classification. Table 2 shows the ASIA classifications and the changes in classification preoperatively, postoperatively, and at final follow-up. When compared to the preoperative ASIA classification, the patients with neurologic deficits including sensory and motor dysfunction of lower limbs were improved by at least one grade. Out of 18 patients with neurological deficits, 15 were recovered to normal status. As shown in Table 2, the average VAS score was 6.4 ± 1.8 preoperatively, decreased to 2.2 ± 0.7 (t = 17.2, *P* < 0.001) before discharge, and further decreased to 0.6 ± 0.6 (t = 20.5, *P* < 0.001) at the final follow-up. The average ODI score was 47.7 ± 12.6 preoperatively, decreased to 25.1 ± 9.0 (t = 18.6, *P* < 0.001) before discharge, and further decreased to 13.1 ± 7.2 (*t* = 22.5, *P* < 0.001) at the final follow-up.

**Table 2:** Characteristics and clinical data of the patients

Time	VAS	odi (%)	ASIA, n		
			C	D	E
Preoperative	6.4 ± 1.8	47.7 ± 12.6	4	14	14
Before discharge	2.2 ± 0.7	25.1 ± 9.0	2	9	21
At final follow-up	0.6 ± 0.6	13.1 ± 7.2		3	29
T/P	17.2/< 0.001^a^	18.6/< 0.001^a^			
	20.5/< 0.001^b^	22.5/< 0.001^b^			

^a^preoperative value compares with preoperative one

^b^preoperative value compares with final follow-up one

As shown in Table 3, the preoperative Cobb angle was 33.6 ± 9.3°. The Cobb angle became 10.6 ± 2.6° (t = 14.4, *P* < 0.001) postoperatively, and was 11.4 ± 3.1° (t = 14.0, *P* < 0.001) at the final follow-up. The mean angle correction was 23.0 ± 8.9°, and the correction rate was 66.2 ± 12.2%. The mean angle lost was 0.8 ± 0.9°, and the loss rate was 5.8 ± 5.4% at the final follow-up. The X-ray and CT scan for all the patients were examined to assess the fusion at the final follow-up. All patients achieved bone fusion. According to the classification of Tan et al, [24] twenty-eight patients achieved complete fusion (Grade I), whereas only four patients achieved partial fusion (Grade II). Grade I denotes the cortical union of the allograft and trabecular continuity between vertebral body and allograft, and Grade II shows the cortical union of the allograft but partial trabecular continuity between vertebral body and allograft.

**Table 3:** Cobb angle, angle correction and angle lost

Characteristics	Descriptive statistics
Preoperative Cobb angle (°)	33.6 ± 9.3
Cobb angle before discharge (°)	10.6 ± 2.6
Angle correction (°)	23.0 ± 8.9
Correction rate (%)	66.2 ± 12.2
Cobb angle at final follow-up (°)	11.4 ± 3.1
Angle lost (°)	0.8 ± 0.9
Lost rate (%)	5.8 ± 5.4
T/P	14.4/< 0.001^a^
	14.0/< 0.001^b^

^a^preoperative value compares with postoperative one

^b^preoperative value compares with final follow-up one

## Discussion

Patients with thoracic and thoracolumbar spinal tuberculosis often shows spinal instability, spinal cord decompression and spinal deformity due to bone destruction and paravertebral or intraspinal abscess. Most patients with spinal tuberculosis can be cured by conservative treatment. Many patients with spinal cord dysfunction can be treated conservatively as effectively as with the urgent early surgical treatment, even patients with tuberculosis of the craniovertebral junction can achieve complete clinical and radiological healing [25, 26]. The surgical management options for spinal tuberculosis remain controversial. However, the surgical treatment to clear tuberculous foci, sclerotic bone, multiple cavities, and bony bridges had been reported to increase curative effect in spinal tuberculosis [27]. Patients treated conservatively have potential to end up with serve deformity and spinal cord compression [28]. The purpose of the surgery of thoracic and thoracolumbar spinal tuberculosis is to relieve spinal cord or nerve compression, to reconstruct the spine stability and remove the vertebral lesion and obvious abscess thoroughly [29–31]. The aim of this study is to evaluate the efficacy and feasibility of allograft bone using titanium mesh and the bony fusion in the posterior-only surgical treatment of thoracic and thoracolumbar spinal tuberculosis.

Various surgical managements have been performed on the patients with spinal tuberculosis patients, including anterior surgery, posterior surgery, and combined anterior-posterior surgery. It had been well recognized that the posterior surgery plays a crucial role in the spinal tuberculosis management. Zhou et al. [5] reported that compared to combined anterior-posterior surgery for the treatment of thoracic and thoracolumbar spinal tuberculosis, posterior surgery could achieve a similar curative effect, and was associated with the advantage of shorter operation time, less blood loss, and shorter hospital stay. The posterior surgical approach is considered better for the reconstruction of spinal stability and the correction of kyphosis [32]. The advantages of posterior surgery include minimum surgical trauma, satisfactory pain relief, excellent neurological recovery, and reconstruction of the spinal stability [21]. Posterior-only surgical approach in spinal tuberculosis is effective in correcting the kyphosis and has achieved satisfactory clinical efficacy. Many researches articles have mentioned the advantages of posterior surgical approach focused on the clinical characteristics, including operation time, blood loss, length of hospital stay, reconstruction of spine stability, neurological recovery, and kyphosis correction. The reconstruction of spine stability and the correction of kyphosis in the posterior surgical approach depends on the posterior fixation devices and the anterior implantation materials, including autogenous or allogeneic bone with or without cages or titanium mesh. When it comes to bony fusion, the distinction of different implantation materials has not been elaborated in detail.

In our study, like previous studies, all patients achieved pain relief after the surgery and at the final follow-up. The average VAS score and the average ODI score decreased after surgery and at the final follow-up. Compared with preoperative scores, the VAS and ODI scores were significantly improved before discharge and at the final follow-up (*P* < 0.001). Moreover, neurological status improved after posterior-only surgical management. Postoperative and follow-up ASIA classification showed visible improvement, which suggests good efficacy and feasibility of posterior-only management for thoracic and thoracolumbar tuberculosis in the improvement of clinical symptoms.

Even though the surgical management of spine tuberculosis are previously studied, the outcome of intervertebral fusion and the reconstruction of large bony defects remain controversial in the posterior-only surgical approach. The methods to reconstruct anterior bony defects include autologous bone (rib or iliac bone), allograft bone grafts, and titanium mesh with autologous or allograft bone particles. One-stage surgical treatment via a posterior-only approach with appropriately sized autologous bone or allograft bone block only is effective and feasible for the treatment of spinal tuberculosis [33]. Therefore, compared with autogenous iliac bone grafts, titanium mesh cages could serve as a superior material in posterior-only surgical approach for thoracic and lumbar spinal tuberculosis [34]. Satisfactory bony fusion and reconstruction of spinal stabilization using titanium mesh with autogenous or allogeneic bone have been well defined. Various findings suggested the advantages of using titanium mesh in patients with osteoporosis and poor iliac bone quality: minor surgical invasion, less complications, effective reconstruction of large defects, and ideal sagittal alignment in lumbosacral tuberculosis [20]. It has been reported that one-stage posterior interbody autogenous graft using titanium mesh cages achieved satisfactory bone fusion in the aged patients with lumbosacral spinal tuberculosis [21]. Ukunda et al. [32] demonstrated that the posterior-only approach using cortical allografts for anterior column reconstruction produced good clinical and radiological outcomes. Titanium mesh with autologous or allograft bone particles in the posterior surgery could construct the bony defects effectively [20]. However, the detailed fusion of allograft bone using titanium mesh in the posterior-only surgery for thoracic and thoracolumbar spinal tuberculosis has not been investigated separately.

In our study, we retrospectively analyzed the patients with thoracic and thoracolumbar spinal tuberculosis who were treated with one-stage posterior debridement, allograft bone graft using titanium mesh, posterior instrumentation, and fusion. We have preferred the allograft using titanium mesh in order to avoid additional autograft harvesting with potential complications. According to previous studies, titanium mesh provide better anterior column support and lower kyphosis angle loss rate [11, 20, 21, 32, 35–37]. Similarly, this study has also concluded that the correction of kyphosis was achieved with satisfactory postoperative cobb angle and acceptable loss of correction. The correction rate and kyphosis angle loss rate as other evaluation indicators for the posterior fixation effectiveness and the anterior bony fusion rate were also summarized. Luo et al. [5] reported a 62.4% correction rate and a 5.5% loss rate of the kyphosis angle in 25 thoracic and thoracolumbar spinal tuberculosis patients who were treated with the posterior approach. Comparing with this study, similar results were concluded in our study. All patients in our study used titanium mesh with allograft to reconstruct the anterior bony defects. All patients achieved bone fusion, including twenty-eight patients with complete fusion (Grade I) and four patients with partial fusion (Grade II), as evaluated by the X-ray and CT images. Loss of correction was not significant in the patients with partial fusion. No graft fracture, infection, or resorption was observed.

The small sample size, the lack of a control group, and study in a single center are the limitations of the study. In conclusion, however, allograft bone using titanium mesh in the posterior-only surgical treatment of thoracic and thoracolumbar spinal tuberculosis achieved good bony fusion according to the radiological evaluation. The satisfactory fusion indicates that allograft bone using titanium mesh can be applied successfully in the spinal tuberculosis patients, especially in patients with osteoporosis and poor iliac bone quality.

## Conclusions

Allograft bone using titanium mesh in the posterior-only surgical treatment is effective management for the patients with thoracic and thoracolumbar spinal tuberculosis. It results in achieve good bony fusion and satisfied correction of kyphosis.

## Data Availability

All data used by or generated in this study is available from the corresponding author upon reasonable request.

## Abbreviations

ASIA: The American Spinal Injury Association
VAS: The visual analog scale
ODI: Oswestry disability index
CT: Computerized tomography
MRI: Magnetic resonance imaging
ESR: The erythrocyte sedimentation rate
CRP: C-reactive protein
